# Evaluating the Effects of Different Vegetation Types on Necrophagous Fly Communities (Diptera: Calliphoridae; Sarcophagidae): Implications for Conservation

**DOI:** 10.1371/journal.pone.0164826

**Published:** 2016-10-31

**Authors:** José Roberto Pereira de Sousa, Fernando da Silva Carvalho-Filho, Leandro Juen, Maria Cristina Esposito

**Affiliations:** 1 Department of Chemistry and Biology, Centro de Estudos Superiores de Imperatriz, Universidade Estadual do Maranhão, Imperatriz-MA, Brazil; 2 Department of Zoology, Coordenação de Zoologia - Entomologia, Museu Paraense Emílio Goeldi, Belém-PA, Brazil; 3 Biological Sciences Institute, Universidade Federal do Pará, Belém-PA, Brazil; USDA Agricultural Research Service, UNITED STATES

## Abstract

The present study was conducted in five different phytogeographic zones of the Brazilian state of Maranhão, three of which (the Amazon Forest, Cerrado, and Palm Groves) are more heterogeneous, whereas the other two (Marshlands and Mangroves) are more homogeneous. In each zone, nine sites were visited for the collection of necrophagous flies using bait traps in 2010, 2011, and 2012. The calliphorid and sarcophagid communities observed at each site were compared in terms of species richness, composition, and abundance. The more heterogeneous zones had higher species richness, except in the case of the sarcophagids in the forest habitats. The calliphorids *Chloroprocta idioidea* (Robineau- Desvoidy, 1830), *Mesembrinella bicolor* (Fabricius, 1805), *Hemilucilia semidiaphana* (Rondani, 1850) and *Lucilia eximia* (Wiedemann, 1819) were more closely associated with the Cerrado, Palm Grove and Amazon Forest zones, and *Chrysomya megacephala* (Fabricius, 194) with the Mangrove. In the sarcophagids, *Peckia* (*Euboettcheria*) *subducta* (Lopes, 1935) and *P*. (*Pattonella*) *palidipilosa* (Curran & Walley, 1934) were associated with the Amazon Forest, and *P*. (*Sarcodexia*) *lambens* (Wiedemann, 1830) and *Tricharaea* (*Sarcophagula*) *occidua* (Fabricius, 1794) with the Palm Grove and Cerrado zones. In the calliphorids, the greatest dissimilarity was recorded between the Amazon Forest and the Mangrove and Lowland grassland zones. In the sarcophagids, by contrast, the greatest dissimilarities were recorded between the Amazon Forest and all the other four zones. In general, then, the phytogeographic zones with the highest environmental heterogeneity were characterized by the greatest species richness and abundance of necrophagous flies.

## Introduction

The type of vegetation found in a given area plays a fundamental role in habitat structure, given that the variation in the height, density, and distribution of trees within the environment creates new conditions and habitats with a larger number of subdivisions. This diversity of conditions is considered to be one of the primary determinants of variation in the species richness levels found within different environments [1–11].

A number of different organisms, especially vertebrates [7], tend to occur at higher densities in structurally more complex environments, in which species with distinct ecological needs can coexist [12, 13]. The environmental heterogeneity hypothesis predicts that animal species richness will tend to increase in more complex environments, where a broader range of niches are available for the different species [14]. More spatially heterogeneous environments may accommodate a larger number of species because they encompass a larger number of microhabitats and a broader range of microclimates, as well as a greater abundance of refuges from predators [15].

In the case of invertebrates, by contrast, and especially for insects, the evidence indicates that patterns of species distribution and diversity are related primarily to the type of vegetation [9], the degree of shade and exposure [16, 17], and temperature and humidity [16]. These features may determine the occurrence of different animal species in a given habitat, depending on how they favor behaviors such as reproduction, nest-building, development, and foraging [12, 18, 19].

The influence of the heterogeneity of the landscape on the spatial distribution of species has been investigated in some dipteran groups [11, 20 – 22]. The levels of habitat disturbance, substrate cover, and the height of the vegetation all had a significant influence on the distribution of Chironomidae species [21]. In the Empididae, the use of different habitats by different species indicates that they respond to the environmental heterogeneity in different ways, and may thus be sensitive to different spatial scales [20]. In drosophilid flies, differences in the species composition between different environments (Cerrado savanna and gallery forest) were related to climatic stability and the greater environmental heterogeneity of the gallery forest [11, 22].

The dipterans of the families Calliphoridae and Sarcophagidae are necrophagous, widely distributed, and occur in a wide diversity of environments [23– 26]. Many of these flies are forensically useful, due to the fact that their larvae feed on dead organic matter, acting as decomposers and providing a potential estimate of the postmortem interval [27]. The adults are also potential vectors of pathogens [28], while the larvae of some species parasitize humans and other vertebrates, causing myiasis, a condition known as blowfly strike [29 – 31].

Both families include species associated with forest environments and more open types of vegetation, as well as urban and rural environments [32, 33]. Studies in Argentina indicated that sites of intermediate impact had a higher diversity of calliphorids and sarcophagids than those where disturbance was either intense or totally absent [34]. Research on calliphorids in the Brazilian Amazon Forest, Atlantic Forest and Mangroves [35– 40] indicates that the greatest variation in species composition and abundance is found between areas with different degrees of forestation, with the highest species richness being found in the forested environments. In sarcophagids, in contrast, the highest species richness in found typically in clearings and other open areas rather than forest [41, 42]. Given this, the type of vegetation and the heterogeneity of the environment appear to be key factors determining the characteristics of calliphorid and sarcophagid communities.

The Brazilian state of Maranhão encompasses a large area located strategically at the interface of the Amazon, Cerrado, and Caatinga biomes [43]. The vegetation of the state reflects the transition from the semi-arid climate of the Brazilian Northeast to the more humid climates of the northern region [44, 45], which comprises enormous diversity of phytophysiognomies, with five main phytogeographic zones—Amazon Forest, Palm Groves (*Mata de cocais*), Cerrado, Marshlands, and Mangroves [43, 46]. The vegetation of these zones varies considerably, with major differences in the physical structure of the environment [47], which varies according to plant species richness and abundance [7]. These zones can be divided into two main types, the more heterogeneous zones, that is, the Amazon Forest [48– 50], Cerrado savannas [51– 54] and Palm Groves [55, 56], and the more homogeneous zones, characterized by a reduced plant diversity, that is, the Marshlands [46] and the Mangroves [57,58].

Given this basic difference, this study evaluated the structure of the necrophagous dipteran communities of the Calliphoridae and Sarcophagidae families in the five phytogeographic zones of the Brazilian state of Maranhão. The study tested the hypothesis that the more heterogeneous phytogeographic zones (Amazon Forest, Cerrado and Palm Groves) are characterized by a greater species richness and abundance of these dipterans than found in the Marshlands and Mangroves.

## Material and Methods

### Study area

**Fig 1:**
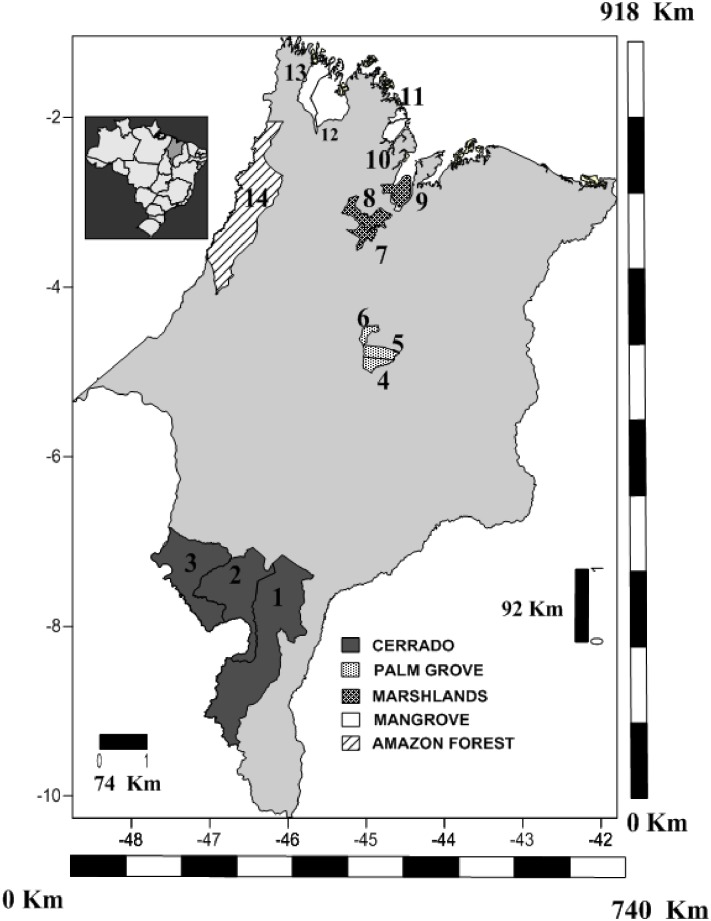
a: Location in Brazil; b: Location of the municipalities in the Brazilian state of Maranhão (modified from Sousa et al. 2015 [60]), in which the samples were collected and their respective phytogeographic zones (Cerrado, Amazon Forest, Palm Groves, Marshlands and Mangrove), sampled between 2010 and 2012. (Key: 1 –Balsas; 2 –Riachão; 3 –Carolina; 4 –Esperantinópolis; 5 –Poção de Pedras; 6 –Lago do Junco; 7 –Cajari; 8 –Viana; 9 –Cajapió; 10 –Guimarães; 11 –Cedral; 12 –Turiaçú; 13 –Cândido Mendes; 14 –Centro Novo do Maranhão. This map was prepared using “shapefile” data from the Brazilian Institute of Geography and Statistics—IBGE (www.ibge.gov.br).

The present study was conducted in 45 areas of natural vegetation located in 14 municipalities of the Brazilian state of Maranhão (Fig 1, Table 1). There are two types of climate found in the state, according to the Köppen classification system, type *Am* in the western extreme (Amazon Forest Zone), which is characterized by a tropical monsoon type of climate with a short dry season, while the *Aw* type prevails in the rest of the state (Cerrado, Palm groves, Marshlands and Mangrove), characterized as a tropical savanna climate, with dry winters and intense summer rains [59].

**Table 1:** Geographic coordinates of the area surveyed in the Brazilian state of Maranhão (2010–2012) and their respective municipalities and phytogeographic zones.

Area	Municipality	South	West	Phytogeographic zones	Number of traps by area
1	Carolina	07°22'10.80"	047°15'56.93"	Cerrado—C	5
2	Carolina	07°20'11.28"	047°15'59.87"	Cerrado—C	5
3	Carolina	07°21'21.60"	047°12'46.00"	Cerrado—C	5
4	Riachão	07°25'28.79''	046°38'51.17''	Cerrado—C	5
5	Riachão	07°42'53.85''	046°45'4.53''	Cerrado—C	5
6	Riachão	07°38'22.85''	046°45'53.15''	Cerrado—C	5
7	Balsas	07°26'44.07''	046°08'29.99''	Cerrado—C	5
8	Balsas	07°28'28.75''	046°06'03.23''	Cerrado—C	5
9	Balsas	07°29'09.59''	046°07'20.99''	Cerrado—C	5
10	Esperantinopolis	04°55'09.02''	044°57'09.43''	Palm Grove—PG	5
11	Esperantinopolis	04°56'04.83''	044°58'07.58''	Palm Grove—PG	5
12	Esperantinopolis	04°55'25.86''	044°55'56.33''	Palm Grove—PG	5
13	PoçãodePedras	04°49'10.95''	044°55'35.55''	Palm Grove—PG	5
14	PoçãodePedras	04°48'5.55''	044°54'06.22''	Palm Grove—PG	5
15	PoçãodePedras	04°46'23.22''	044°53'00.38''	Palm Grove—PG	5
16	LagodoJunco	04°36'51.45''	045°02'26.62''	Palm Grove—PG	5
17	LagodoJunco	04°35'56.37''	045°02'01.28''	Palm Grove—PG	5
18	LagodoJunco	04°33'23.03''	045°03'04.09''	Palm Grove—PG	5
19	Cajari	03°19'18.03''	044°52'13.06''	Marshlands—ML	5
20	Cajari	03°18'49.3''	044°54'26.08''	Marshlands—ML	5
21	Cajari	03°17'10.0'	044°57'21.01''	Marshlands—ML	5
22	Cajapió	02°51'33.5''	044°41'31.08''	Marshlands—ML	5
23	Cajapió	02°51'12.6''	044°42'46.06''	Marshlands—ML	5
24	Cajapió	02°49'07.67''	044°44'31.22''	Marshlands—ML	5
25	Viana	03°14'16.2''	044°56'25.3''	Marshlands—ML	5
26	Viana	03°12'51.6''	045°02'19.5''	Marshlands—ML	5
27	Viana	03°12'21.59''	,045°03'51.25''	Marshlands—ML	5
28	Cedral	01°57'44.3'	044°30'41.6''	Mangrove—M	5
29	Cedral	01°59'13.79''	044°29'41.21''	Mangrove—M	5
30	Cedral	02°00'20.2''	044°34'20.2''	Mangrove—M	5
31	Guimaraes	02°03'50.54''	044°31'05.21''	Mangrove—M	5
32	Guimaraes	02°02'54.2''	044°29'47.7''	Mangrove—M	5
33	Guimaraes	02°02'11.37''	044°33'03.09''	Mangrove—M	5
34	Cândido Mendes	01°25'54.39''	045°30'45.12''	Mangrove—M	5
35	Cândido Mendes	01°29'30.67''	045°32'39.54''	Mangrove—M	5
36	Turiaçú	01°31'07.4''	045°25'47.2''	Mangrove—M	5
37	Centro Novo do MA	03°37'09.1''	046°43'22.6''	Amazon Forest—AF	5
38	Centro Novo do MA	03°35'47.34''	046°42'54.56''	Amazon Forest—AF	5
39	Centro Novo do MA	03°36'34.6''	046°44'20.8''	Amazon Forest—AF	5
40	Centro Novo do MA	03°35'39.8''	046°45'48.3''	Amazon Forest—AF	5
41	Centro Novo do MA	03°33'57.90''	046°45'32.78''	Amazon Forest—AF	5
42	Centro Novo do MA	03°33'35.14''	046°50'17.11''	Amazon Forest—AF	5
43	Centro Novo do MA	03°35'34.60''	046°50'25.40''	Amazon Forest—AF	5
44	Centro Novo do MA	03°32'07.16''	046°47'39.95''	Amazon Forest—AF	5
45	Centro Novo do MA	03°34'52.4''	046°46'43.6''	Amazon Forest—AF	5

The Cerrado vegetation is a heterogeneous mosaic of habitats, with grasslands at one extreme and forest formations at the other, forming a gradient of tree height and density [61, 62]. Based on the size and density of its trees and shrubs, the Cerrado can be divided into four habitat subgroups—savanna woodland (Cerradão), tree savanna, park savanna and grassy-shrubby savanna [63]. Savanna woodland areas (Fig 1) were selected for this study. This is a forest formation with a non-grassy herbaceous stratum dominated by seedlings and shrubs, with relatively well-developed tree cover and taller trees than those found in the other savanna subtypes [64].

The predominant vegetation in the Amazon Forest is dense rainforest (Fig 1), which is the region’s most exuberant type of habitat, with trees reaching 20 m in height [63]. Tropical forests are known for their high plant diversity [65], which is sustained in large part by niche differentiation [66] and is closely related to the spatial heterogeneity of the forest.

The Palm Groves are located between the Cerrado and the Amazon Forest, and are characterized by a mixture of plant species found in these vegetation types (Fig 1). This forest is evergreen with prevailing babaçu palm (*Orbignya phalerata*), and trees of reduced height in comparison with the typical dense Amazon broadleaf forest.

The Marshland zone (Fig 1) is an immense area formed by a series of lakes with extensive marshes and flooded grassland, which are dry during approximately seven months of the year. These floodplains are dominated by open vegetation, with several taller trees and palms, in addition to the igapó swamp, found at the banksof streams, rivers, and lakes, lasting four to six months of the year (Fig 1). The areas selected for this study were open fields dominated by herbaceous vegetation interspersed with a few trees and lianas.

The mangrove is a microphanerophyte community found in brackish environments, generally at the mouths of rivers and in coastal channels, where the muddy soils support a specialized vegetation adapted to highly saline conditions and include: *Rhizophora mangle* L., *Avicennia* spp. (the local species depends on its latitude), and *Laguncularia racemosa* L. which grows on the highest terrain and is only flooded at high tide (Fig 1). One or two of these elements may be missing from some areas, however, a homogeneous *Rhizophora* mangrove is typical of some parts of the Maranhão coast [63].

### Data collection

The permits for the collection and transportation of zoological specimens (necrophagous Diptera) were provided by the Chico Mendes Institute for the Conservation of Biodiversity (ICMBio) / Sisbio, in accordance with federal law and the regulations of the Brazilian Environmental Ministry, through process number 1403–1 (for private properties) and 29342–1 (for the conservation unit, the Gurupi Biological Reserve in the state of Maranhão). In private sampling sites, permission from the owner or manager was obtained prior to sampling. None of the sampled species were protected by Brazilian law or red-listed.

The campaigns for the collection of fly specimens were conducted during the dry season, between August and October in 2010, April through October in 2011, and May through November in 2012.

The 45 areas (sampling units) were distributed evenly among the five phytogeographic zones, three of which (Cerrado, Amazon Forest and Palm Groves) are more heterogeneous in terms of the complexity of their vegetation, whereas the other two zones (Marshlands and Mangrove) were more homogeneous. Nine well-conserved areas (replicates) were sampled in each zone, always in natural habitats, with a minimum distance of 2 km between sites. Each area was sampled twice, with five traps per area, making a total of 225 traps, with 450 samples (two per trap) being collected by the end of the study.

The specimens were collected in traps designed specifically for the capture of saprophagous dipterans [32], as described by Almeida et al. [67] and used successfully by Sousa et al [37]. The traps were baited with 50 g of cow lung, and were placed in shaded locations, along a 1 km transect at intervals of 200 m, for 48 h. A total of 45 traps were set in each of the nine phytogeographic zones.

The calliphorid specimens were identified using the keys of Mello [68], Carvalho & Mello-Patiu [69], and Kosmann et al. [70]. The sarcophagids were identified using the species keys available for the genera *Thexysarcodexia* Townsend [71, 72] and *Peckia* Robineau-Desvoidy [73] and other references, including the studies of Lopes [74– 77], Tibana [78,79], Tibana & Xerez [80], and Guimarães [81].

Part of the material collected was prepared in a dry medium and deposited in the Entomology Collection of the Goeldi Museum (MPEG) and the Zoology Museum at the Biological Sciences Institute of UFPA, both in Belém. The rest of the specimens were conditioned in liquid medium (70% ethanol) and then included in the teaching collection of Prof. Clésio Fonseca Zoology Laboratory at the Imperatriz campus of Maranhão State University.

### Data analysis

The species richness was estimated for each phytogeographic zone using a first-order jackknife approach [82] run in EstimateS, version 9.0 [83]. The sampling efficiency was evaluated through cumulative species curves, using the same estimator, with 1,000 randomizations based on the number of traps [84]. Richness estimators were used to evaluate the heterogeneity of the data. Each occurrence of a species considered rare [83] increases the heterogeneity of the dataset and the probability of encountering a new species. In this case, the first-order jackknife estimator is less strict than other estimators, with species occurring in only one sample ("unique species") being considered rare [85], and was thus considered the most appropriate estimator for this study. We emphasize that we also performed the analyses using the first-order Chao estimator in addition to the jackknife estimate, but the result obtained using the two estimators were congruent. Therefore, we chose to present the article only the results of the first-order jackknife. A confidence interval-based inference approach was used to test the hypothesis that the differences found among the phytogeographic zones influenced the species richness of calliphorids and sarcophagids. This approach was also based on first-order jackknife estimates [84], with the zones being considered significantly different when their respective confidence intervals did not overlap the mean values of the other zone.

Species composition was analyzed using non-metric multidimensional scaling (NMDS) based on a Bray-Curtis dissimilarity matrix [86, 87], with the data being log (x+1) transformed to reduce the effect of discrepant values. To test the hypothesis that differences among the phytogeographic zones influence the composition and abundance of calliphorid and sarcophagid species, a nonparametric permutational analysis of variance (PERMANOVA) for models with multiple factors was applied, based on a Bray-Curtis similarity index, with 9,999 permutations [88]. When significant results were obtained for the PERMANOVA, multiple pairwise *a posteriori* tests were applied for comparisons between zones.

The mean similarity of the zones based on the structure of the calliphorid and sarcophagid community, and the species that most contributed to the similarity among environments were determined by a percentage similarity analysis, or SIMPER [87]. The dissimilarity between zones and the species that most contributed to this dissimilarity were also analyzed using SIMPER [87].

## Results

A total of 6,498 calliphorid specimens were collected, distributed in seven genera and 12 species (Table 2; S1 Table). The most abundant were *Chrysomya albiceps* (Wiedemann) (29.38% of the specimens) and *Cochliomyia macellaria* (Fabricius) (23.07%). A total of 2,921 sarcophgid specimens (Table 3; S2 Table) were collected, representing 11 genera and 40 species, of which the most abundant were *Tricharaea (Sarcophagula) occidua* (Fabricius) (32.80%) and *Peckia* (*Sarcodexia*) *lambens* (Wiedemann) (20.16%).

**Table 2:** Composition and abundance of calliphorid species in the different phytogeographic zones surveyed in the Brazilian state of Maranhão, between 2010 and 2012.

SPECIES	PZ-C	PZ-PG	PZ-AF	PZ-ML	PZ-M	TOTAL	%
*Chrysomya albiceps* (Wiedemann, 1819)	1,270	194	30	109	306	**1,909**	**29.32**
*Chrysomya megacephala* (Fabricius, 1794)	106	54	0	0	578	**738**	**11.33**
*Chrysomya putoria* (Wiedemann, 1819)	34	6	0	0	38	**78**	**1.20**
*Chloroprocta idioidea* (Robineau-Desvoidy,1830)	382	234	426	12	0	**1,054**	**16.19**
*Cochliomyia hominivorax* (Coquerel, 1858)	3	0	1	0	1	**5**	**0.08**
*Cochliomyia macellaria* (Fabricius, 1775)	647	317	22	419	94	**1,499**	**23.02**
*Hemilucilia benoisti* Séguy, 1925	0	1	0	0	0	**1**	**0.02**
*Hemilucilia segmentaria* (Fabricius, 1805)	2	0	1	0	0	**3**	**0.05**
*Hemilucilia semidiaphana* (Rondani, 1850)	6	6	156	0	0	**168**	**2.58**
*Lucilia eximia* (Wiedemann, 1819)	135	540	108	10	0	**793**	**12.18**
*Mesembrinella bicolor* (Fabricius, 1805)	66	0	169	0	0	**235**	**3.61**
*Paralucilia paraensis* (Mello, 1969)	11	3	1	0	0	**15**	**0.23**
**ABUNDANCE**	**2,662**	**1,355**	**914**	**550**	**1,017**	**6,498**	**100**
**ABUNDANCE %**	**40.97**	**20.85**	**14.07**	**8.46**	**15.65**	**100**	
**RICHNESS (S)**	**11**	**9**	**9**	**4**	**5**		

Legend: PZ = Phytogeographic Zones; C = Cerrado; PG = Palm Grove; AF = Amazon Forest; ML = Marshlands; M = Mangrove.

**Table 3:** Composition and abundance of sarcophagid species in the different phytogeographic zones surveyed in the Brazilian state of Maranhão, between 2010 and 2012.

SPECIES	PZ-C	PZ-PG	PZ-AF	PZ-ML	PZ-M	TOTAL	%
*Blaesoxipha* (*Gigantotheca*) *stallengi*	2	0	0	0	0	**2**	**0.07**
*Helicobia aurescens* (Townsend, 1927)	1	1	0	0	0	**2**	**0.07**
*Helicobia borgmeieri* Lopes,1939	1	0	0	0	0	**1**	**0.03**
*Helicobia morionella* (Aldrich, 1930)	6	2	0	0	1	**9**	**0.31**
*Helicobia pilifera* Lopes, 1939	11	0	0	0	0	**11**	**0.38**
*Helicobia pilipleura* Lopes 1939	0	2	0	0	0	**2**	**0.07**
*Helicobia rapax* (Walker, 1849)	0	4	0	0	0	**4**	**0.14**
*Oxysarcodexia admixta* (Lopes, 1933)	0	4	0	0	0	**4**	**0.14**
*Oxysarcodexia amorosa* (Schiner, 1868)	0	0	0	1	15	**16**	**0.55**
*Oxysarcodexia aura* (Hall,1937)	1	0	0	0	0	**1**	**0.03**
*Oxysarcodexia avuncula* (Lopes, 1933)	1	11	0	0	0	**12**	**0.41**
*Oxysarcodexia bakeri* (Aldrich, 1916)	0	0	0	0	1	**1**	**0.03**
*Oxysarcodexia fringidae* (Curran & Walley, 1934)	0	1	0	18	2	**21**	**0.72**
*Oxysarcodexia intona* (Curran & Walley, 1934)	0	213	0	214	28	**455**	**15.58**
*Oxysarcodexia modesta* Lopes, 1946	8	0	0	0	0	**8**	**0.27**
*Oxysarcodexia thornax* (Walker, 1849)	37	43	0	0	0	**80**	**2.74**
*Oxysarcodexia timida* (Aldrich, 1916)	11	1	0	0	11	**23**	**0.79**
*Oxysarcodexia vilosa* Lopes, 1946	0	0	0	0	3	**3**	**0.1**
*Peckia* (*Euboettcheria*) *anguilla* (Curran & Walley, 1934)	3	10	2	0	1	**16**	**0.55**
*Peckia* (*Euboettcheria*) *collusor* (Curran & Walley, 1934)	28	62	5	4	21	**120**	**4.11**
*Peckia* (*Euboettcheria) subducta* (Lopes, 1935)	0	0	1	0	0	**1**	**0.03**
*Peckia* (*Peckia*) *chrysostoma* (Wiedemann, 1830)	67	121	0	83	126	**397**	**13.59**
*Peckia* (*Peckia*) *pexata* (Wulp, 1895)	8	15	0	10	0	**33**	**1.13**
*Peckia* (*Peckia*) *uncinata* (Hall, 1933)	0	0	0	2	0	**2**	**0.07**
*Peckia* (*Pattonella*) *intermutans*(Walker, 1861)	11	3	0	0	0	**14**	**0.48**
*Peckia* (*Pattonella*) *palidipilosa* (Curran & Walley, 1934)	0	0	1	0	0	**1**	**0.03**
*Peckia* (*Squamatodes*) *ingens* (Walker,1849)	39	35	6	0	0	**80**	**2.74**
*Peckia* (*Squamatodes*) *trivitata* (Curran, 1927)	1	1	0	0	0	**2**	**0.07**
*Peckia* (*Sarcodexia*) *lambens* (Wiedemann, 1830)	290	237	13	34	15	**589**	**20.16**
*Ravinia belforti* (Prado & Fonseca, 1932)	1	1	0	0	0	**2**	**0.07**
*Ravinia effrenata* (Walker, 1861)	20	0	0	0	0	**20**	**0.68**
*Retrocitomyia mizuguchiana* Tibana & Xerez, 1985	4	0	0	2	0	**6**	**0.21**
*Retrocitomya uromajoensis* Lopes, 1985	0	0	0	0	5	**5**	**0.17**
*Sarcophaga (Lipoptilocnema) misella* Lopes, 1938	1	0	0	0	0	**1**	**0.03**
*Sarcofahrtiopsis cuneata* Townsend, 1935	2	0	0	0	0	**2**	**0.07**
*Sarcophaga polistensis* Hall, 1933	1	0	0	0	0	**1**	**0.03**
*Titanogrypa* (*Cucullomyia*) *larvicida* (Lopes, 1935)	1	0	0	0	0	**1**	**0.03**
*Tricharaea* (*Sarcophagula*) *canuta* Wulp, 1896	0	0	0	0	13	**13**	**0.45**
*Tricharaea* (*Sarcophagula*) *occidua* (Fabricius, 1794)	864	45	1	18	30	**958**	**32.8**
*Villegasia almeidai* (Lopes, 1938)	0	0	0	0	2	**2**	**0.07**
**ABUNDANCE**	**1,420**	**812**	**29**	**386**	**274**	**2,921**	**100**
**ABUNDANCE %**	**48.61**	**27.8**	**0.99**	**13.21**	**9.38**	**100**	
**RICHNESS (S)**	**26**	**20**	**7**	**10**	**15**		

Legend: PZ = Phytogeographic Zones; C = Cerrado; PG = Palm Grove; AF = Amazon Forest; ML = Marshlands; M = Mangrove.

### Species richness

**Fig 2:**
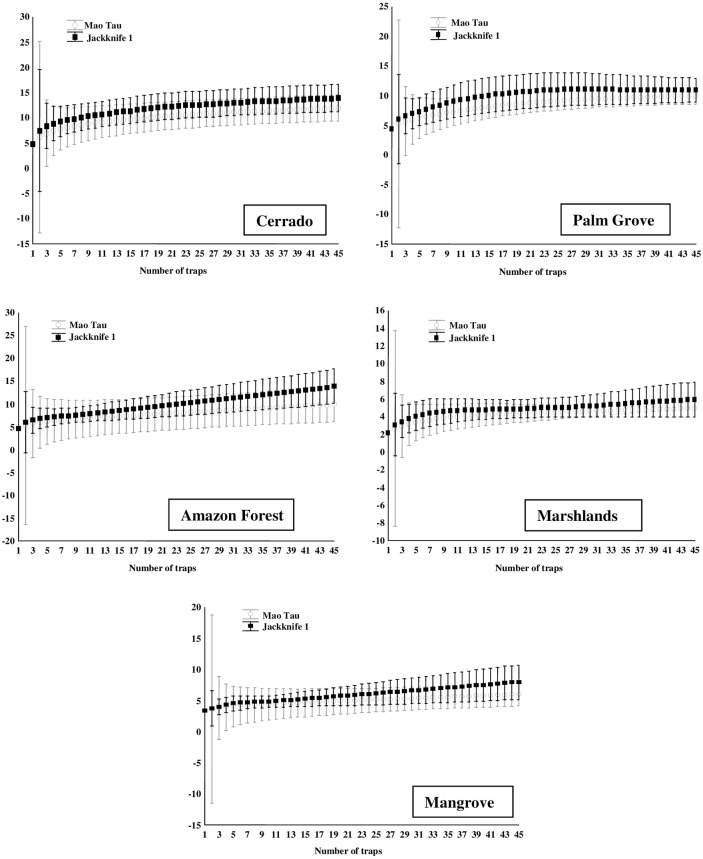
Cumulative species curves (Observed [Mao Tau] and Estimated [Jackknife1] species richness) for the family Calliphoridae (traps as pseudoreplicates) in the different phytogeographic zones surveyed in the Brazilian state of Maranhão between 2010 and 2012. Axis Y: estimated species richness (Jackknife).

**Fig 3:**
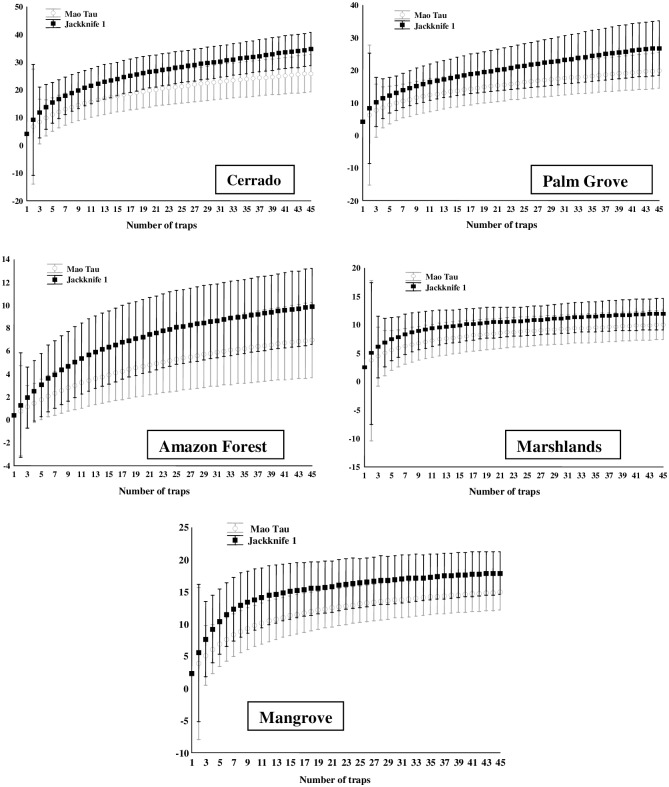
Cumulative species curves (Observed [Mao Tau] and Estimated [Jackknife1] species richness) for the family Sarcophagidae (traps as pseudoreplicates) in the different phytogeographic zones surveyed in the Brazilian state of Maranhão between 2010 and 2012. Axis Y: estimated species richness (Jackknife).

**Fig 4:**
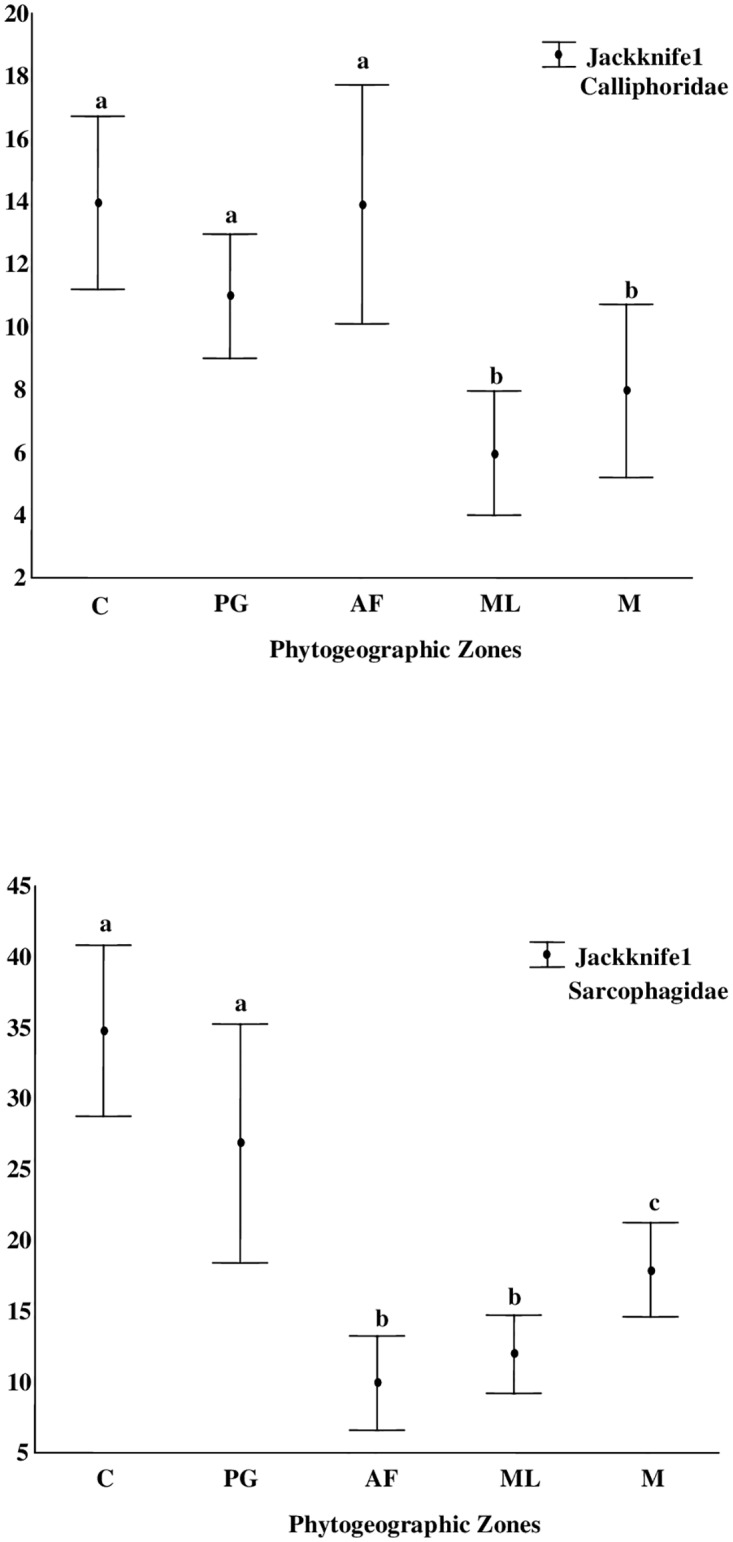
Estimated species richness (mean±confidence interval) of the Calliphoridae and Sarcophagidae (Jackknife 1) in the five phytogeographic zones sampled in the Brazilian state of Maranhão between 2011 and 2012. The values indicated by different letters are significantly different from one another. Legend: C = Cerrado; PG = Palm Grove; AF = Amazon Forest; ML = Marshlands; M = Mangrove. Axis Y: estimated species richness (Jackknife).

The sampling efficiency varied between 63% and 82% for the family Calliphoridae, and between 70% and 83% for the Sarcophagidae, indicating that the sampling effort was sufficient to estimate species richness. In some zones, however, the cumulative species curve did not stabilize (Figs 2 and 3, Table 4). The highest calliphorid species richness was estimated for the Cerrado zone (13.96±2.76), followed by the Amazon Forest (13.91±3.81), and Palm Grove (10.98±1.98), but with no significant difference among the zones (Fig 4). The highest sarcophagid richness was also recorded in the Cerrado (34.8±6.05) and Palm Grove (26.84±8.42), which were statistically similar (Fig 4). The Amazon Forest (9.93±3.33) returned the lowest estimated richness of sarcophagid species for any zone.

**Table 4:** Sampling efficiency (observed and estimated species richness) of the calliphorid and sarcophagid species in the different phytogeographic zones (Cerrado, Palm Grove, Marshlands, Mangroves, and Amazon Forest) surveyed in the Brazilian state of Maranhão between 2010 and 2012.

**Family Calliphoridae**	**PZ-Cerrado**	**PZ-Palm Grove**	**PZ-Amazon Forest**	**PZ-Marshlands**	**PZ-Mangrove**
Observed Richness	11	9	9	4	5
Estimated Richness	14	11	14	6	8
Sampling Efficiency (%)	79	82	64	67	63
**Family Sarcophagidae**	**PZ-Cerrado**	**PZ-Palm Grove**	**PZ-Amazon Forest**	**PZ-Marshlands**	**PZ-Mangrove**
Observed Richness	26	20	7	10	15
Estimated Richness	35	27	10	12	18
Sampling Efficiency (%)	74	74	70	83	83

Legend: PZ = Phytogeographic Zones

### Composition and abundance

Significant differences were found in the composition and abundance of both calliphorid (pseudo-F = 54.01; d.f. = 4; p < 0.001) and sarcophagid (pseudo-F = 6.69; d.f. = 4; p < 0.001) species in the different phytogeographic zones (Table 5). The results were significant (p < 0.01) for all the comparisons among zones, in both the Calliphoridae and the Sarcophagidae (Table 6), which predominate in the Cerrado (40.97% of the Calliphoridae and 48.61% of the Sarcophagidae) and Palm Grove, with 20.85% and 27.80%, respectively (Tables 2 and 3).

**Table 5:** Results of the Permutational Analysis of Variance (PERMANOVA) of the community structure of the calliphorids and sarcophagids recorded in the different phytogeographic zones (Cerrado, Palm Grove, Amazon Forest, Marshlands and Mangrove) of the Brazilian state of Maranhão. d.f. = degrees of freedom.

**PERMANOVA—Calliphoridae**				
**Treatment**	**d.f.**	**F**	**p**	**Contribution**
**Zones**	4	54.01	0.0001	36.18
**Residues**	40			14.9
**PERMDISP—Calliphoridae**	4	6.52	0.0001	
**PERMANOVA—Sarcophagidae**				
**Treatment**	**d.f.**	**F**	**p**	**Contribution**
**Zones**	4	6.69	0.0001	31.95
**Residues**	37			38.72
**PERMDISP -Sarcophagidae**	4	2.97	0.0756	

**Table 6:** Multiple *a posteriori* pairwise comparisons for the results of the Permutational Analysis of Variance (PERMANOVA) of the community structure of the calliphorids and sarcophagids recorded in the five phytogeographic zones of the Brazilian state of Maranhão.

PHYTOGEOGRAPHIC ZONES	Calliphoridae			Sarcophagidae		
	t	P(perm)	perms	t	P(perm)	perms
**Cerrado x Palm Grove**	2.65	0.0005	8123	1.93	0.0008	8166
**Cerrado x Marshlands**	4.9	0.0001	8139	3.60	0.0002	8159
**Cerrado x Mangrove**	5.62	0.0001	8117	2.76	0.0002	8166
**Cerrado x Amazon Forest**	6.8	0.0001	8164	2.66	0.0008	4328
**Palm Grove x Marshlands**	5.67	0.0003	8136	2.47	0.0004	8171
**Palm Grove x Mangrove**	8	0.0001	8154	2.12	0.0006	8192
**Palm Grove x Amazon Forest**	8.22	0.0001	8199	2.58	0.0002	4324
**Marshlands x Mangrove**	5.94	0.0001	8163	1.92	0.0017	8183
**Marshlands x Amazon Forest**	10.34	0.0002	8121	3	0.0001	4314
**Mangrove x Amazon Forest**	16.66	0.0002	8141	2.56	0.0001	4311

**Fig 5:**
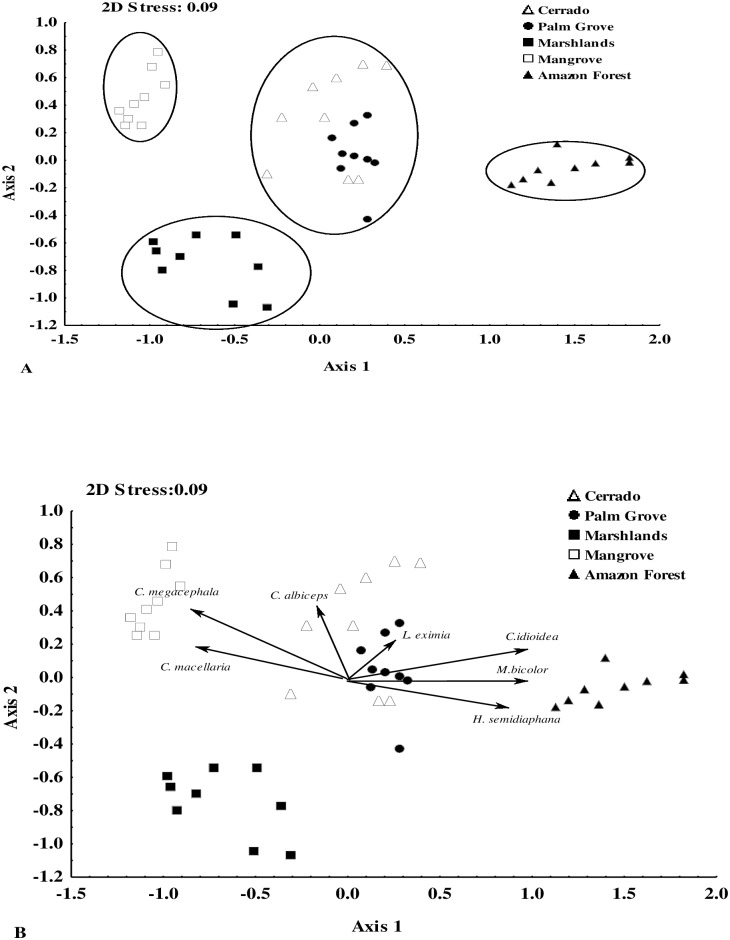
Multi-dimensional non-metric scaling (NMDS) of the 45 study areas representing the five main phytogeographic zones (Cerrado, Palm Grove, Amazon Forest, Marshlands, and Mangrove) found in the Brazilian state of Maranhão, between 2010 and 2012, based on the composition and abundance of calliphorid species. A) Groups defined by the analysis; B) Association of species with the phytogeographic zones.

**Fig 6:**
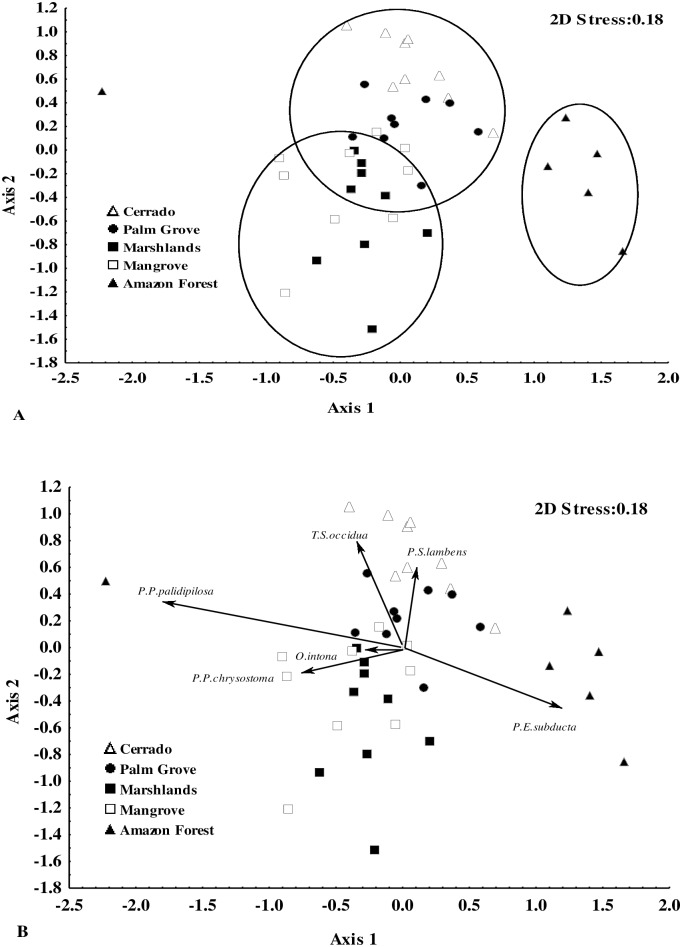
Multi-dimensional non-metric scaling (NMDS) of the 45 study areas representing the five principal phytogeographic zones (Cerrado, Palm Grove, Amazon Forest, Marshlands, and Mangrove) found in the Brazilian state of Maranhão, between 2010 and 2012, based on the composition and abundance of sarcophagid species. A) Groups defined by the analysis; B) Association of species with the phytogeographic zones.

The ordination analysis showed a segregation of the calliphorid and sarcophagid communities among zones. In the calliphorids, there was a clear gradient from the more homogeneous zones (Marshlands and Mangrove) to the most heterogeneous ones, i.e., Amazon Forest, Cerrado, and Palm Grove (Fig 5A; axis 1). In the sarcophagids, however, the Amazon Forest was clearly separated from the other zones (Fig 6A; axis 1).

A strong association was observed between the calliphorids *Chloroprocta idioidea* (Robineau-Desvoid), *Mesembrinella bicolor* (Fabricius), *Hemilicilia semidiaphana* (Rondani) and *Lucilia eximia* (Wiedemann) and the Cerrado, Palm Grove, and Amazon Forest zones, and of *Chrysomya megacephala* (Fabricius) with the Mangrove zone (Fig 5B). Similarly, the sarcophagids *Peckia* (*Euboettcheria*) *subducta* (Lopes) and *Peckia* (*Pattonella*) *palidipilosa* were associated with the Amazon Forest, *P*.(*S*.) *lambens* with the Palm Grove and Cerrado, and *T*. (*S*.) *occidua* with the Cerrado (Fig 6B).

### Similarities among zones

The mean similarity between zones varied from 77% to 86% in the calliphorids, and from 31% to 57% in the sarcophagids (Table 7). The calliphorids *C*. *albiceps*, *L*. *eximia*, *C*. *macellaria*, *C*. *megacephala* and *C*. *idioidea* contributed most to the observed pattern. The sarcophagid species that most contributed to the similarity among zones were *P*. (*S*.) *lambens*, *P*. *(P)*. *chrysostoma* and *Oxysarcodexia intona* (Curran & Walley).

**Table 7:** Similarity of the zones based on the structure of the calliphorid and sarcophagid communities.

Phytogeographic Zones	Calliphoridae				Sarcophagidae			
	% MS	SPECIES	% C	% AC	% MS	SPECIES	% C	% AC
**Cerrado**	74	*C*. *albiceps*	30	30	55	*P*. (*S*.) *lambens*	31	31
		*C*. *macellaria*	24	54		*T*. (*S*.) *occidua*	18	49
		*C*. *idioidea*	22	77		*P*. (*P*.) *chrysostoma*	17	66
**Palm Grove**	83	*L*. *eximia*	27	27	53	*P*. (*P*.) *chrysostoma*	29	29
		*C*. *idioidea*	24	51		*P*. (*S*.) *lambens*	21	50
		*C*. *macellaria*	20	71		*P*. (*E*.) *collusor*	19	68
**Amazon Forest**	86	*C*. *idioidea*	30	30	31	*P*. (*S*.) *lambens*	85	85
		*M*. *bicolor*	23	53		*P*. (*E*.) *anguilla*	6	91
		*H*. *semidiaphana*	22	74				
**Marshlands**	77	*C*. *macellaria*	58	58	57	*O*. *intona*	46	46
		*C*. *albiceps*	38	97		*P*. (*P*.) *chrysostoma*	31	77
						*P*. (*S*.) *lambens*	12	89
**Mangrove**	86	*C*. *megacephala*	41	41	41	*P*. (*P*.) *chrysostoma*	48	48
		*C*. *albiceps*	33	74		*P*. (*E*.) *collusor*	11	59
		*C*. *macellaria*	23	98		*O*. *intona*	8	68

Legend: % MS = Mean Similarity; % C = Contribution of the species to the similarity index. % AC = Accumulated Contribution.

In the case of the calliphorids, *C*. *albiceps* and *C*. *macellaria* were the main species responsible for the mean levels of similarity in the Cerrado and Marshland zones, whereas *C*. *megacephala* and *C*. *albiceps* were the key species for the Mangrove, *L*. *eximia* and *C*. *idioidea* contributed most to the similarity in the Palm Grove, and *C*. *idioidea* and *M*. *bicolor* in the Amazon Forest (Table 6).

In the sarcophagids, the key species contributing to similarity were *P*. (*S*.) *lambens* and *T*. (*S*.) *occidua* in the Cerrado; *P*.(*P*.) *chrysostoma* and *P*. (*S*.) *lambens* in the Palm Grove; *O*. *intona* and *P*. (*P*.) *chrysostoma* in the Marshlands; *P*. (*S*.) *lambens* and *P*. (*Euboettcheria*) *anguilla* (Curran & Walley) in the Amazon Forest; and *P*. (*P*.) *chrysostoma* (Wiedemann, 1830) and *P*. *(Euboettcheria) collusor* (Curran & Walley) in the Mangrove (Table 6).

### Dissimilarity among zones

In the calliphorids, the highest dissimilarity scores were obtained for the comparisons between the Amazon Forest and the Mangrove and Marshland zones. The highest values for the sarcophagids were found between the Amazon Forest and all the other four zones, in other words, the Palm Grove, Cerrado, Marshlands, and Mangrove (Table 8).

**Table 8:** Dissimilarity between zones (significant results of the *a posteriori* pairwise tests of the PERMANTOVA) based on the structure of the calliphorid and sarcophagid communities.

Phytogeographic Zones	Calliphoridae				Sarcophagidae			
	% MD	Espécie	% C	% AC	% MD	Espécie	% C	% AC
**Cerrado x Palm Grove**	29	*L*. *eximia*	19	19	54	*T*. (*S*.) *occidua*	15	15
		*C*. *albiceps*	19	38		*O*. *intona*	12	27
		*C*. *megacephala*	12	50		*P*. (*S*.) *lambens*	10	37
**Cerrado x Marshlands**	49	*C*. *idioidea*	23	23	70	*O*. *intona*	16	16
		*C*. *albiceps*	17	40		*P*. (*S*.) *lambens*	14	30
		*L*. *eximia*	14	55		*T*. (*S*.) *occidua*	13	44
**Palm Grove x Marshlands**	47	*L*. *eximia*	33	33	58	*O*. *intona*	15	15
		*C*. *idioidea*	25	58		*P*. (*S*.) *lambens*	14	29
		*C*. *megacephala*	16	74		*P*.(*E*.) *collusor*	13	42
**Cerrado x Mangrove**	46	*C*. *idioidea*	26	26	71	*P*. (*S*.) *lambens*	17	17
		*C*. *megacephala*	16	42		*T*. (*S*.) *occidua*	14	30
		*L*. *eximia*	15	57		*P*. (*S*.) *ingens*	8	38
**Palm Grove x Mangrove**	49	*L*. *eximia*	30	30	64	*P*. (*S*.) *lambens*	14	14
		*C*. *idioidea*	25	55		*O*. *intona*	12	27
		*C*. *megacephala*	18	73		*P*. (*S*.) *ingens*	10	37
**Marshlands x Mangrove**	47	*C*. *megacephala*	48	48	60	*O*. *intona*	20	20
		*C*. *macellaria*	16	64		*P*. (*P*.) *chrysostoma*	12	32
		*C*. *albiceps*	12	76		*P*. (*S*.) *lambens*	10	43
**Cerrado x Amazon Forest**	53	*C*. *albiceps*	20	20	81	*P*. (*S*.) *lambens*	17	17
		*C*. *macellaria*	17	38		*T*. (*S*.) *occidua*	17	35
		*H*. *semidiaphana*	15	52		*P*. *(P*.*) chrysostoma*	13	48
**Palm Grove x Amazon Forest**	48	*M*. *bicolor*	21	21	80	*P*. (*P*.) *chrysostoma*	20	20
		*H*. *semidiaphana*	18	39		*P*. (*S*.) *lambens*	14	34
		*C*. *macellaria*	16	54		*O*. *intona*	14	48
**Marshlands x Amazon Forest**	74	*C*. *idioidea*	21	21	86	*O*. *intona*	31	31
		*M*. *bicolor*	19	40		*P*. (*P*.) *chrysostoma*	22	53
		*H*. *semidiaphana*	18	58		*P*. (*S*.) *lambens*	11	65
**Mangrove x Amazon Forest**	85	*C*. *megacephala*	19	19	88	*P*. (*P*.) *chrysostoma*	25	25
		*C*. *idioidea*	18	37		*P*. (*E*.) *collusor*	10	36
		*M*. *bicolor*	14	51		*O*. *intona*	9	45

Legend: % MD = Mean Similarity; % C = Contribution of the species to the similarity index. % AC = Accumulated Contribution.

## Discussion

The calliphorid and sarcophagid communities varied considerably among the different phytogeographic zones found in the Brazilian state of Maranhão. The communities in the more structured and heterogeneous vegetation types had higher species richness than those observed in the less complex zones, with the exception of the sarcophagids in the Amazon Forest zone.

The lower sarcophagid species richness recorded in the Amazon Forest may be related to microclimatic variables and the dense vegetation characteristics of this environment, which may be unfavorable to many species that depend on exposure to sunlight [89– 91]. In a number of studies, larger numbers of sarcophagid flies were collected on bait exposed in sunny areas in comparison with more shaded sites [91]. Furthermore, adults were observed flying or visiting flowers during the sunniest hours of the day [89, 90]. The color of the abdominal cuticle of the sarcophagids has a high capacity for thermal reflectance [90], which could help minimize overheating. Sousa and colleagues [42] also recorded a lower species richness of sarcophagids in forests, in comparison to neighboring environments, with varying degrees of vegetation cover (clearings at different stages of recuperation) in the Amazon Forests.

In the case of the calliphorids, studies in the Brazilian Atlantic Forest [38–40, 92, 93] and Amazonia [35–37] have invariably recorded higher species richness in forests in comparison to open habitats. This positive relationship between species richness and habitat complexity appears to be typical of these dipterans [7], and is based on the premise that a more complex environment will provide a greater abundance of potential niches, enabling the coexistence of a larger number of species [3]. For the calliphorids, in particular, the more complex formations (Cerrado, Amazon Forest, and Palm Grove) may provide a greater diversity of potential feeding resources for both adults and larvae, such as feces and animal carcasses, as well as providing more shaded and stable environments. The resources available in open environments are more exposed to rainfall and dehydration, which makes them more ephemeral. As the sarcophagids are viviparous or ovoviviparous [94], their larvae remain in the substrates where they breed for shorter periods, and they are therefore less exposed to these environmental conditions. In this case, less sarcophagid diversity was lost in the more open formations (mangrove and marshlands) than in the more complex habitats, in contrast with the pattern observed in the calliphorids. However, sarcophagid diversity was also higher in the more complex and heterogeneous environments, such as the Cerrado and palm groves, which are naturally more open than the Amazon Forest. This may also account for the lower abundance and species richness of sarcophagids found in the Amazon Forest, where the incidence of sunlight is limited by the closed canopy, restricting the occurrence of many species.

The calliphorid and sarcophagid communities were distributed along a vegetation complexity gradient (homogenous–heterogeneous), with patterns of occurrence and abundance typical of each zone. The species *C*. *idioidea*, *M*. *bicolor* and *H*. *semidiaphana* and *L*. *eximia*, which were associated with the Amazon Forest, Cerrado and Palm Grove, have already been recorded in forest environments [35– 37, 39,95] and *C*. *megacephala*, which was associated with the Mangrove zone, has been registered in the mangroves of the Brazilian state of Rio de Janeiro [40,96]. *Peckia* (*Euboettcheria*) *subducta* (Lopes) and *Peckia* (*Pattonella*) *palidipilosa* (Curran & Walley) were associated with the Amazon Forest, *P*. (*S*.) *lambens* with the Palm Grove and Cerrado zones, and *T*. (*S*.) *occidua* the Cerrado.

The distribution pattern of the calliphorids resulted in greater mean similarity among the areas surveyed in each zone than the sarcophagids. This appears to be related to the much greater species diversity of the Sarcophagidae, which is double that of the Calliphoridae [97]. The Calliphoridae is known to include 1525 species, while the Sarcophagidae has close to 3094 [97]. While only 130 calliphorid species are found in the Neotropical region [98], there are about 800 sarcophagids [99], and in Brazil, only 38 calliphorid species have been recorded [100] compared with 350 sarcophagids [101]. These differences alone would be enough to account for the greater similarities found within the group with the lowest diversity (i.e., the Calliphoridae). It is important to note that the restriction of a species to forested areas may filter its distribution in an extremely selective way, although it may also result in a wider niche for other factors, such as feeding and egg-laying substrates, which would favor niche overlap, with no competitive exclusion of species. The calliphorid species that most contributed to the similarity found within each zone included species associated with more forested and well-preserved habitats, such as *C*. *idioidea* and *L*. *eximia* [32, 33, 37, 102], as well as those associated with more open and urbanized environments, like *C*. *albiceps*, *C*. *megacephala* and *C*. *macellaria* [103]. The sarcophagids *P*. (*S*.) *lambens*, *P*. (*P*.) *chrysostoma* and *O*. *intona* have been recorded in both forests and more open or urbanized environments [104].

The greatest dissimilarities in calliphorid diversity were found between the most complex and heterogeneous zone (forest) and the most homogeneous ones (Marshlands and Mangroves). In the case of the sarcophagids, however, the greatest dissimilarities were found between the forests and all other zones, including the Cerrado and the Palm Grove. This indicates the influence of some additional factor that affects the diversity of sarcophagids in Amazon forested habitats, probably related to microclimatic factors and the greater shading in these environments, which reduces the potential exposure of the flies to sunlight. Given this, the selection of areas for the conservation of these and other taxa, should consider not only the contribution of the structurally more complex habitats, but areas representative of all the biomes, including the more homogeneous ones, such as the mangroves and Marshlands.

Our results have shown that the natural environments found in the different phytogeographic zones are inhabited by distinct sets of species. The composition of the fly communities varies because of the different climatic conditions of the distinct zones and the characteristics of their plant cover, which combine to create a unique set of environmental conditions under which the species survive, reproduce, and interact. These differences reinforce the need to take into account the characteristics of each distinct area when planning the conservation of a given region. Of the five vegetation types sampled in this study in the state of Maranhao, only the Cerrado and the Amazon rainforest have full conservation protection units, in other words, those with a higher degree of restriction of use by man (Cerrado 3%, Amazon Forest 2%) [43]. Furthermore, given the differences observed in the present study, and the local history of land use for farming and ranching, there is also an urgent need for the investigation of the effects of anthropogenic impacts on the calliphorid and sarcophagid communities found in each phytophysiognomy. These data will be essential for a more systematic understanding of the conservation status of the two groups within each study area.

## Supporting Information

S1 Table Full list of dipteran necrophagous fauna of Calliphoridae family, sampling locations and environments. (XLS)

S2 Table Full list of dipteran necrophagous fauna of Sarcophagidae family, sampling locations and environments. (XLS)
